# Investigation of genetic variants regulating the expression of metabotropic glutamate receptor, subtype 5 (GRM5) mRNA in human brain

**DOI:** 10.1186/1750-1326-7-S1-S23

**Published:** 2012-02-07

**Authors:** Jian Wang, David Saffen

**Affiliations:** 1 Institutes of Brain Science, Fudan University, Shanghai 200032, P.R. China; 2 State Key Laboratory for Medical Neurobiology, Fudan University, Shanghai 200032, P.R. China; 3 School of Life Science, Fudan University, Shanghai 200032, P.R. China

## Background

The metabotropic glutamate receptor, subtype 5 (GRM5) regulates cell excitability and neurotransmission and has been suggested to play a modifying role in several neuropsychiatric and neurodegenerative disorders, including Parkinson’s disease (PD). The observation that GRM5 inhibitors ameliorate symptoms and slow the degradation of nigrostriatal dopamine neurons in rodent models of PD suggests that low expression of GRM5 may also reduce symptoms in human patients and, possibly, slow the development of the disease. The goal of the present study is quantify common variation of *GRM5* mRNA expression in human brain and identify haplotypes or combinations of genotypes that correlate with mRNA expression. Haplotypes and genotype combinations that predict mRNA expression should be useful as markers in genetic association studies aimed at detecting possible contributions of *GRM5* to PD and other disorders.

## Methods

Sixty-three independent frozen sections of prefrontal cortex (Han Chinese; Brodmann area 46) were obtained from the China Brain Bank Center (Wuhan). Genomic DNA and total RNA were isolated using standard procedures. A common SNP, rs566277 (heterozygosity = 0.42), located within the 3’-untranslated region of *GRM5* mRNA was chosen as a molecular marker to distinguish mRNAs derived from each autosomal allele. SNaPShot^©^-based AEI assays were carried out as previously described [Lim J *et al*., *Molecular Psychiatry*, 2007]. Analysis of population distributions of log_2_AEI ratios was carried out using a mathematical model developed in-house. Levels of *GRM5* mRNA relative to mRNA encoding the house-keeping gene GAPDH were quantified using real-time PCR (calculated as ΔCt). Genomewide genotyping of samples was carried out using HumanOmni1-Qad arrays (Illumina). SNPs for which allelic heterozygosity and homozygosity correlate with AEI were identified by calculating Kappa coefficients for single-, double-, and triple- combinations of SNPs within a 500,000 base pair region of chromosome 11 centered on *GRM5*. SNPs with genotypes that correlate with *GRM5* mRNA expression were identified by one-way ANOVA analysis for SNPs in same region.

## Results

Eleven of 18 samples heterozygous for the marker SNP showed robust differential expression between alleles. Mathematical modeling of the population distribution of log_2_AEI ratios suggested that *GRM5* mRNA is regulated by two *cis*-acting genetic elements that are partially linked to the marker SNP (D’ = 0.24) and tightly linked with each other (D’ = 1.0). Three sets of tightly linked SNPs located in immediate the 5’-upstream region (rs7120151 + rs12421343 or rs4378401 or rs496939) were found to fit this model (r-squared ~ 0.9 for predicted *vs* experimentally determined log_2_AEI population distributions) and to be highly correlated with AEI (Kappa coefficient = 0.75 for rs7120151 + rs12421343 or rs4378401, and 0.5 for rs7120151 + rs496939). Correlations between genotype and relative *GRM5* mRNA expression for rs7120151 and rs496939 just missed statistical significance (P = 0.05 – 0.057), but were statistically significant for the tightly linked SNPs rs655683 and rs7126679 (P = 0.035 and 0.03).

## Conclusions

Our study suggests that expression of *GMR5* mRNA is regulated by two tightly linked genetic elements located within a 77 kb region centered on the exon 1 and the promoter region. SNPs that highly correlate with AEI and mRNA expression should provide useful markers in genetic association studies aimed at evaluating the potential role of *GRM5* as a genetic modifier of neuropsychiatric and neurodegenerative disorders, including PD.

